# Different latent class models were used and evaluated for assessing the accuracy of campylobacter diagnostic tests: overcoming imperfect reference standards?

**DOI:** 10.1017/S0950268818001723

**Published:** 2018-06-27

**Authors:** J. Asselineau, A. Paye, E. Bessède, P. Perez, C. Proust-Lima

**Affiliations:** 1 Bordeaux University Hospital, Public Health Department, Clinical Epidemiology Unit, F-33076 Bordeaux, France; 2 INSERM, CIC 1401 Clinical Epidemiology, F-33076 Bordeaux, France; 3 French National Reference Center for Campylobacter and Helicobacter, F-33076 Bordeaux, France; 4 INSERM, UMR1219, Univ. Bordeaux, ISPED, F-33076 Bordeaux, France

**Keywords:** Campylobacter, diagnostic accuracy, imperfect gold standard, latent class model, sparseness

## Abstract

In the absence of perfect reference standard, classical techniques result in biased diagnostic accuracy and prevalence estimates. By statistically defining the true disease status, latent class models (LCM) constitute a promising alternative. However, LCM is a complex method which relies on parametric assumptions, including usually a conditional independence between tests and might suffer from data sparseness. We carefully applied LCMs to assess new campylobacter infection detection tests for which bacteriological culture is an imperfect reference standard. Five diagnostic tests (culture, polymerase chain reaction and three immunoenzymatic tests) of campylobacter infection were collected in 623 patients from Bordeaux and Lyon Hospitals, France. Their diagnostic accuracy were estimated with standard and extended LCMs with a thorough examination of models goodness-of-fit. The model including a residual dependence specific to the immunoenzymatic tests best complied with LCM assumptions. Asymptotic results of goodness-of-fit statistics were substantially impaired by data sparseness and empirical distributions were preferred. Results confirmed moderate sensitivity of the culture and high performances of immunoenzymatic tests. LCMs can be used to estimate diagnostic tests accuracy in the absence of perfect reference standard. However, their implementation and assessment require specific attention due to data sparseness and limitations of existing software.

## Introduction

The evaluation of new diagnostic tests requires the assessment of the diagnostic test accuracy, usually in terms of sensitivity and specificity. It consists in confronting the index test results with the presence/absence of the target condition. The reference standard is the best method available for attesting the target condition [1]. However, in many cases it is not a Gold Standard, it does not have sensitivity and specificity of 100%. This imperfection of the reference standard translates into the misclassification of patients with regard to the target condition and entails biased estimates of the prevalence and accuracy of new diagnostic tests [2–7]. Different solutions have been proposed and reviewed to evaluate diagnostic test accuracy in the presence of an imperfect Gold standard as the correction of accuracy parameters according to external data or the use of multiple reference standards (expert panel, composite reference standard) [4, 5]. An alternative is to rely on a purely statistical approach, the latent class framework, sometimes referred to as latent class models (LCM), latent class analyses (LCA) or mixture models [8–16]. This method defines the true target condition status as two latent classes and estimates of the sensitivities and specificities of the diagnostic tests, possibly including the imperfect Gold standard, according to these latent classes. LCMs have sparked a growing interest in Biostatistics with contributions evaluating their robustness to the misspecification of the assumption of independence between diagnostic tests conditionally on the true condition status [17–21], recommending methods to assess this assumption [10, 14, 22–24] and proposing extensions to take into account such misspecification [10, 12, 25, 26].

In the recent years, clinical applications of this approach have been reported in the medical and veterinary literature. However, the models and the underlying assumptions or application conditions were rarely evaluated whereas such violations can lead to biased reported estimates of diagnostic accuracy [14].

Diarrhoeal diseases are responsible for 550 million people falling ill yearly, including 220 million children under the age of 5 years and campylobacter bacteria is the most frequent cause of bacterial gastroenteritis worldwide. Campylobacter infections are generally mild but can be fatal among very young children, elderly and immunosuppressed individuals [27]. Its diagnosis relies on stool cultures that have a moderate sensitivity because of the fragility and special culture requirement of campylobacter bacteria (microaerobic environment): a too long contact with a not controlled atmosphere may inhibit the growth of the bacteria. By contrast, specificity is expected to be very high as it may almost only be altered by bacterial contamination. As campylobacter growth is also slow and usually takes more than 48 h, other diagnostic tests (immunoenzymatic, molecular and immunochromatographic methods) have been developed and are available. They are easy to apply and interpret and their results are obtained much more quickly than those of culture (from 30 minutes to 2 h). However, their diagnostic accuracy cannot be correctly assessed using conventional comparisons due to the imperfect reference standard of culture [28–30]. For example, true cases missed by culture but detected by the new test would be incorrectly classified as false positives. By accounting for the imperfection of culture, LCMs, by contrast, have the potential to correctly estimate the diagnostic accuracy of these new tests. In addition, LCMs can take into account a conditional dependence between tests, which arises for example when different tests make the same error. In campylobacter diagnosis, it might be the case when considering various immunological tests, especially if based on the same campylobacter antigens.

In this work, we aimed at carefully applying the LCM methodology to evaluate diagnostic test accuracy through a real case study, the evaluation of new rapid diagnostic tests of campylobacter. Based on current recommendations [14] and statistical developments, we specifically explored the means to assess goodness of fit of LCMs, mostly regarding the sparseness of the data and the violation of the conditional independence assumption and to implement extended LCMs involving random effects.

## Methods

### Study population

Our analysis relies on data from two studies that included every stool specimen obtained from a patient with a gastrointestinal illness at Bordeaux University Hospital (Bordeaux, France) from June to October 2009 and at Lyon University Hospital (Lyon, France) from February to September 2012 [28, 31]. Stools were sent to the laboratory at room temperature without transport medium. The fresh, unpreserved stools were tested for culture within 4 h after arriving at the laboratory. The remaining part of the stool samples was then frozen at −80 °C. The other tests were performed together, once a week, after the samples were thawed. For the Lyon study, culture and immunochromatographic tests were performed in Lyon, ELISAs and polymerase chain reaction (PCR) were performed in Bordeaux.

### Diagnostic tests

Every stool specimen was tested by five different diagnostic tests for campylobacter briefly described below. For more details, please refer to Bessède *et al.* [28]:

**−** *Culture*. A stool suspension was prepared, plated on a Karmali agar (Oxoid, Basingstoke, Hampshire, UK) and the plates were incubated for a maximum of 3 days in a microaerobic atmosphere. Colonies resembling campylobacter colonies were tested with a MALDI-TOF mass spectrometer.
**−** *Rapid immunochromatographic tests*. ImmunoCardSTAT!Campy (Meridian Bioscience, Inc., Cincinnati, OH, USA) was used according to the manufacturer's instructions. It is an immunochromatographic test which detects specific campylobacter antigens on a band. The result was read and validated if the control line band was clearly visible.
**−** *ELISAs*. Two different tests were used: RIDASCREEN^®^ Campylobacter (R-Biopharm AG) and Premier^®^ Campy (Meridian Bioscience, Inc.), both according to the manufacturer's instructions.
**−** *Real-time PCR*. The real-time PCR and hybridisation reactions were performed according to the method published by Ménard *et al.*, using a LightCycler thermocycler (Roche Diagnostics, Meylan, France) [32].

### Statistical analysis

We considered different specifications of LCM to estimate diagnostic accuracy parameters (sensitivity, specificity, negative and positive predictive values) of each test and the prevalence of campylobacter infection in the study population.

#### Standard LCM

The LCM assumes that the true target condition status, campylobacter infection in our case, is not observed. It is statistically defined by a binary latent variable with two modalities corresponding to the absence and the presence of the disease (called disease-free latent class and disease latent class, respectively). The disease prevalence is thus given by the probability *P* to belong to the disease latent class. Sensitivity Se_*k*_ and specificity Sp_*k*_ of each test *k* (*k* = 1,…,*K*) correspond to the conditional probabilities of each test result given the latent classes.

**Fig. 1.**
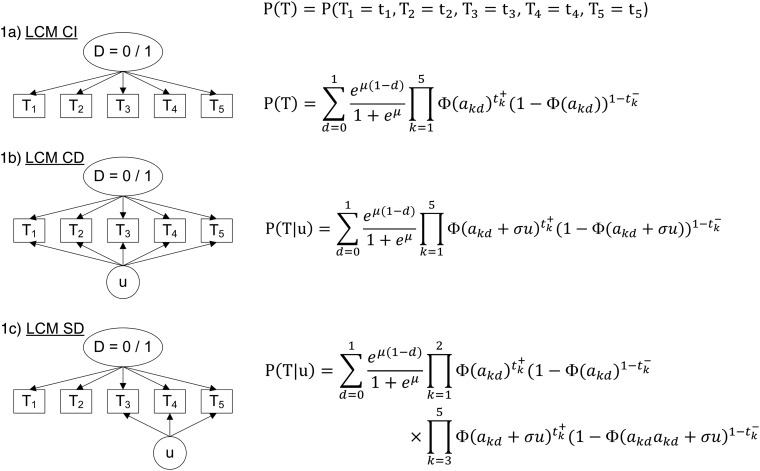
Diagram (left panel) and corresponding profile probability (right panel) for three latent class models assuming different dependence structures, CampyLCA study, France, 2016. LCM CI, latent class model under conditional independence; LCM CD, latent class model with a residual dependence common to all tests; LCM SD, latent class model with a residual dependence specific to the three immunoenzymatic tests. Ovals and rectangles indicate latent quantities and observed quantities, respectively: *D* = 0/1: unobserved presence/absence of campylobacter infection; *T*_1_: Culture Karmali; *T*_2_: Real-time PCR; *T*_3_: Ridascreen^®^; *T*_4_: Premier^®^Campy^®^; *T*_5_: ImmunoCardStat!^®^Campy; u: random residual dependence which follows a standard Gaussian distribution. In the equations, *t*_*k*_+ and *t*_*k*_− indicate a positive and negative result for test *T*_*k*_, respectively; Φ is the standard cumulative Gaussian distribution function; parameters to estimate are (a_kd_)*k* = 1,…,*K*, *d* = 0, 1 for the probit transformations of sensitivities and specificities, *μ* for the logit transformation of the prevalence and *σ* for the intensity of the individual random deviation.

The standard LCM (LCM CI, Fig. 1a) relies on the central assumption, called ‘conditional independence assumption’, that test results are independent given the latent classes. Based on this assumption, the probability of observing a response profile for the *K* tests denoted *T*_1_ to *T*_*K*_ can be expressed as the sum of two terms, one per class:

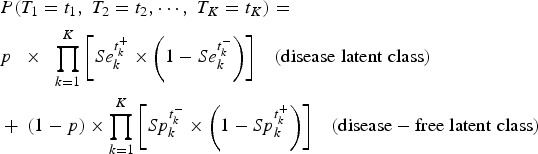
 with *t*_*k*_ the result of test *T*_*k*_ noted 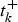 if positive and 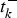 if negative.

In estimation procedures, Se_*k*_ and Sp_*k*_ probabilities (for *k* = 1, …, *K*) are usually modelled using a probit link while the prevalence *p* is usually modelled using a logit link [10, 12].

#### Extended LCMs

The conditional independence assumption is rarely verified in practice so we investigated two main alternatives which consider residual dependences between tests through individual random effects [10, 12]:

**−** A LCM with a common residual dependence between the tests (LCM CD, Fig. 1b). In this model, an individual random intercept, added to the probit model for the sensitivities and specificities of the tests, captures the residual correlation between tests.
**−** A LCM with a specific residual dependence within the three immunoenzymatic tests (Premier^®^Campy, Ridascreen^®^, ImmunoCard Stat!^®^Campy) (LCM SD, Fig. 1c). In this model, an individual random intercept is added to the probit models only for a subset of tests suspected to be conditionally dependent.

#### Estimation of standard and extended LCMs

Parameters of LCMs were estimated in the maximum likelihood framework. Identifiability of LCMs requires that the number of parameters does not exceed 2^*K*^–1 (2^*K*^ being the number of possible dichotomous tests combinations). So, at least three tests in LCM CI and four tests in LCM CD and SD are necessary to estimate all the parameters (2K sensitivity/specificity parameters, plus one for prevalence plus one for the intensity associated with the random intercept in LCM CD and LCM SD). We implemented the models using RandomLCA package [33] and NLMIXED procedure (SAS Institute, Inc., SAS software version 9.3, Cary, North Carolina). Because convergence towards local maxima is frequently encountered in mixture models [34], we considered 100 sets of random initial values, either completely at random (with R) or chosen within clinically plausible ranges (with SAS) to ensure convergence towards the global maximum. The introduction of a random effect in the likelihood for LCM CD and LCM SD induced an integral that had to be numerically solved [35]. It was done with adaptive Gaussian quadratures implemented in both programs. After convergence, two-sided 95% confidence interval (95% CI) of each parameter was obtained by a Monte Carlo approximation.

#### A posteriori evaluation of LCM

As recommended by some authors [10, 12, 14, 23, 24], we used a series of post-fit criteria and posterior analyses to thoroughly assess the models:

(1) Models were compared in terms of Akaike Information Criterion.
(2) Absence of residual dependence between the tests was verified using goodness-of-fit statistics which compare model predictions with observations (Pearson, Likelihood Ratio and Power Divergence statistics). Both the asymptotic Chi-square distribution and empirical distributions were considered for the statistic under the null hypothesis. Indeed, in the context of sparse data (many profiles with low frequencies), results with the asymptotic distribution may not apply and only those obtained with empirical distributions are recommended [12, 22–24, 36]. The empirical distribution was obtained by generating a large number of samples (*n* = 500) from the null assumption and computing the corresponding statistic; the *P*-value was deduced from the quantile which corresponded to the statistic in the observed sample.
(3) Pairwise residual correlations and bivariate residual statistics were calculated to detect potential residual dependences between pairs of diagnostic tests that were not correctly taken into account [10, 12, 24, 37].
(4) Leave-one-test-out analyses were performed by removing one by one each immunoenzymatic test in order to assess their influence on the diagnostic accuracy of the other medical tests [14].

In supplementary simulations, we assessed the type-I error rates obtained with the asymptotic distributions of statistics from point 2 in the specific case of our application data to better appreciate the performances of usual statistics in our sparseness context. The type I error rate quantifies the percentage of times when the test concludes that observations and predictions significantly differ while they actually do not. We also evaluated the power of the same statistics relying on the empirical distributions to detect a violation to the conditional independence assumption. The power quantifies the percentage of times when the test concludes that observations and predictions significantly differ and they actually do.

Statistical tests were all performed at the significance level of 5%.

## Results

From the 32 profiles of test responses possible with five dichotomous diagnostic tests (2^5^), 17 were observed and only 10 included at least three patients (Table 1) among the 623 patient samples. This underlines the sparseness of our data. The most frequent profiles were ‘all tests negative’ (83.8%) and ‘all tests positive’ (6.6%). According to the classical reference standard, bacteriological culture, the prevalence of campylobacter infection was 9.0% (95% CI 8.8–9.3).

**Table 1.** Test results profiles: observed and predicted (by the Latent Class Models) number of patients for each combination of test results, CampyLCA Study, France, 2016

Culture Karmali	Real-time PCR	Rida-screen^®^	Premier^®^ Campy	ImmunoCard Stat!^®^ Campy	Observed patients (%)		Predicted patients		
							LCM CI	LCM CD	LCM SD
−	−	−	−	−	522	(83.8)	519.6	521.2	522.1
−	−	−	−	+	15	(2.4)	16.9	16.4	15.6
−	−	−	+	+	4	(0.6)	0.2	0.7	1.4
−	−	+	−	−	7	(1.1)	9.6	7.6	5.2
−	−	+	−	+	3	(0.5)	0.4	1.8	4.1
−	−	+	+	+	2	(0.3)	2.6	2.4	3.0
−	+	−	−	−	3	(0.5)	3.4	2.4	3.0
−	+	+	−	−	1	(0.2)	0.1	0.5	0.2
−	+	+	+	−	1	(0.2)	1.7	1.9	1.2
−	+	+	+	+	9	(1.4)	10.8	7.1	8.7
+	−	−	−	−	2	(0.3)	1.9	1.5	2.0
+	−	−	+	−	1	(0.2)	0.1	0.2	0.0
+	−	+	+	−	1	(0.2)	1.2	1.2	0.8
+	−	+	+	+	5	(0.8)	8.1	4.3	5.4
+	+	+	−	−	1	(0.2)	0.2	0.2	0.8
+	+	+	+	−	5	(0.8)	5.2	3.5	5.7
+	+	+	+	+	41	(6.6)	34.2	42.4	40.2

LCM CD, latent class model with a residual dependence common to all tests; LCM CI, latent class model under conditional independence; LCM SD, latent class model with a residual dependence specific to the three immunoenzymatic tests.

Predicted profiles frequencies estimated with the three LCMs were globally close to observed frequencies (Table 1) but the two models considering a residual dependence (LCM CD and LCM SD) were closer at least for the most frequent profiles. For instance, the ‘all negative tests’ profile was predicted at 519.6, 521.2 and 522.1 with LCM CI, CD and SD., respectively, for 522 patients observed; similarly, the ‘all tests positive’ profile was predicted at 34.2, 42.4 and 40.2 with LCM CI, CD and SD, respectively, for 41 patients observed. Note that among the 15 non-observed profiles, two profiles with LCM CI and CD model and one profile for LCM SD had predicted frequencies above one (data not shown).

Comparison of LCM CI, CD and SD models in terms of goodness of fit is summarised in Table 2. We only interpret in the following the statistics based on empirical distributions due to the sparseness of our data; results based on the asymptotic distributions are given only to illustrate their lack of reliability in the presence of sparseness. LCM CI under conditional independence hypothesis presented the worst Akaike information criterion and this specification was highly rejected by all statistics. The LCM CD provided an improved Akaike information criterion (by 17.6 points) but all the test statistics still rejected the adequacy of the model. LCM SD provided the best Akaike information criterion (improved by 29.6 points compared with LCM CI and 12.0 compared with LCM CD) and none of the goodness-of-fit tests rejected the specification of LCM SD even if *P*-values were just above the significance threshold. We explored other dependency structures but the latter, based on biological knowledge, remained the most satisfying one.

**Table 2.** Akaike information criterion and goodness-of-fit statistics for each model, CampyLCA Study, France, 2016

	LCM CI	LCM CD	LCM SD
Akaike information criterion	1041.5	1023.9	1011.9
Pearson statistics			
Asymptotic χ^2^ distribution	<0.001	0.012	0.021
Empirical distribution^a^	<0.001	0.022	0.052
Likelihood ratio statistics			
Asymptotic χ^2^ distribution	<0.001	0.052	0.52
Empirical distribution^a^	<0.001	0.004	0.086
Power divergence statistics			
Asymptotic χ^2^ distribution	<0.001	0.039	0.20
Empirical distribution^a^	<0.001	0.012	0.054

LCM CD, latent class model with a residual dependence common to all tests; LCM CI, latent class model under conditional independence; LCM SD, latent class model with a residual dependence specific to the three immunoenzymatic tests.

a The *P*-value when using the empirical distribution was calculated as one minus the percentile of the statistic in 500 samples generated under the null assumption.

**Fig. 2.**
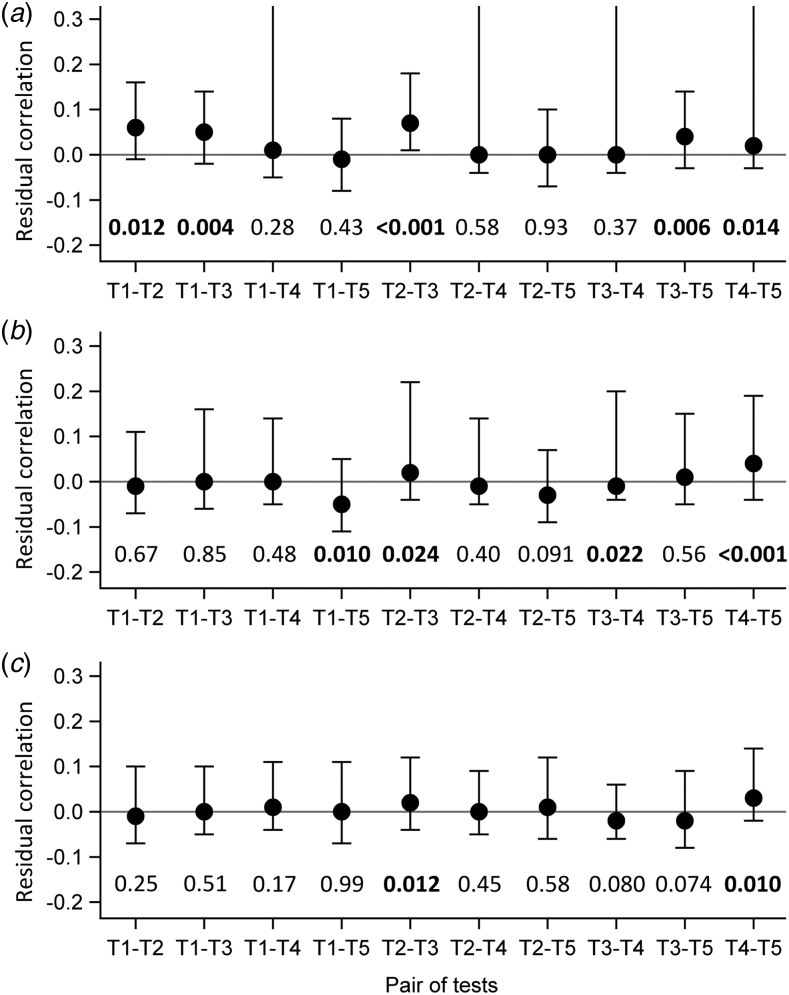
Evaluation of local independence hypothesis by residual correlations and their 95% confidence interval, as well as by *P*-values of bivariate statistics, CampyLCA study, France, 2016. (a) Residual correlations for latent class model under conditional independence; (b) Residual correlations for latent class model with a residual dependence common to all tests; (c) Residual correlations for latent class model with a residual dependence specific to the three immunoenzymatic tests. *T*_1_: Culture Karmali; *T*_2_: Real-time PCR; *T*_3_: Ridascreen^®^; *T*_4_: Premier^®^Campy; *T*_5_: ImmunoCard Stat!^®^Campy. Residual correlations presented with dots (point estimates) and bars (95% confidence intervals). *P*-values of bivariate statistics are provided above each pair of tests described on the horizontal axis.

Evaluation of the local independence hypothesis is shown in Figure 2. While residual correlations were not highlighted for LCM CD or SD based on their 95% confidence interval, bivariate statistics rejected the local independence hypothesis at the 5% level for four pairs of tests in LCM CD and still for two pairs of tests for LCM SD.

**Fig. 3.**
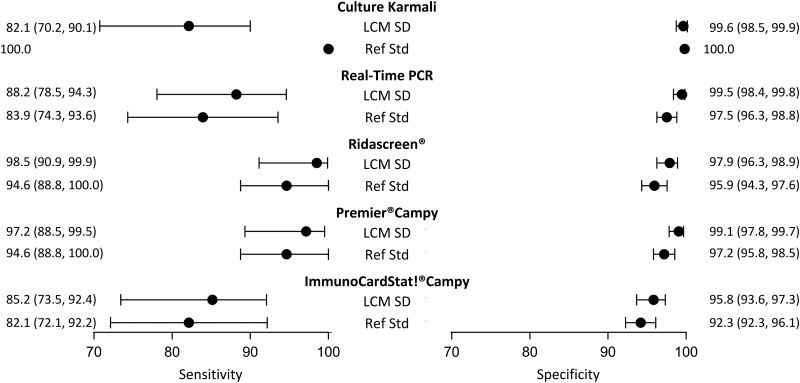
Diagnostic accuracy estimates (point estimate and 95% confidence interval) of campylobacter infection tests according to the LCM SD model and to culture as the reference standard, CampyLCA study, France, 2016. LCM SD, latent class model with a residual dependence specific to the three immunoenzymatic tests; Ref Std: culture Karmali.

Although it did not satisfy all the criteria, the LCM SD model presented the best evaluations. According to this model (Table 3), the prevalence of campylobacter infection was estimated at 10.5% (95% CI 8.4–13.3). The standard error of the random effect specific to the three immunoenzymatic tests was statistically different from zero (estimated at 1.6, (95% CI 0.9−2.4)). The diagnostic accuracy of the different medical tests according to LCM SD are displayed and compared with those obtained when using the culture as the reference standard in Figure 3. As expected, culture presented a moderate sensitivity (82.1%, (95% CI 70.2–90.1)). Using culture as the reference standard resulted in a systematic underestimation of other tests sensitivities and specificities. According to LCM SD model Ridascreen^®^ and Premier^®^Campy tests showed the best compromise between sensitivity and specificity, both above 97%. Estimations and 95% CI of accuracy parameters of all medical tests according to all the LCMs are given in Table 3.

**Table 3.** Diagnostic accuracy of medical tests according to LCM models, CampyLCA Study, France, 2016

	Reference standard		LCM CI		LCM CD		LCM SD	
	Sensitivity, 95% CI							
Culture Karmali	100.0		76.0	64.7–84.8	78.4	64.8–88.4	82.1	70.7–90.0
Real-time PCR	83.9	74.3–93.5	80.9	70.5–89.0	83.9	67.8–93.3	88.2	78.1–94.6
Ridascreen^®^	94.6	88.7–100.0	93.0	85.3–97.2	96.7	68.7–99.9	98.5	91.1–99.9
Premier^®^Campy	94.6	88.7–100.0	97.1	87.0–99.7	96.9	83.4–99.7	97.2	89.3–99.5
ImmunoCard Stat!^®^Campy	82.1	72.1–92.2	86.7	76.7–93.2	85.8	74.7–93.1	85.2	73.4–92.1
	Specificity, 95% CI							
Culture Karmali	100.0		99.6	98.7–99.9	99.5	98.3–99.9	99.6	98.7–99.9
Real-time PCR	97.5	96.3–98.8	99.4	98.1–99.8	99.3	98.0–99.8	99.5	98.4–99.8
Ridascreen^®^	95.9	94.3–97.6	98.2	96.7–99.1	98.2	96.6–99.1	97.9	96.3–98.9
Premier^®^Campy	97.2	95.8–98.5	100.0	NE	99.5	95.4–100.0	99.1	97.8–99.7
ImmunoCard Stat!^®^Campy	94.2	92.3–96.1	96.9	95.2–98.1	96.4	94.1–97.8	95.8	93.7–97.3
	Negative predictive value, 95% CI							
Culture Karmali	100.0		97.0	95.2–98.2	97.4	95.2–98.7	97.9	96.4–98.9
Real-time PCR	98.4	97.4–99.4	97.6	96.0–98.7	98.1	95.6–99.3	98.6	97.3–99.4
Ridascreen^®^	99.5	98.8–100.0	99.1	98.0–99.6	99.6	95.8–100.0	99.8	98.9–100.0
Premier^®^Campy	99.5	98.8–100.0	99.6	0.0–99.9	99.6	97.9–100.0	99.7	98.7–99.9
ImmunoCard Stat!^®^Campy	98.2	97.0–99.3	98.3	96.8–99.2	98.2	96.6–99.2	98.2	96.7–99.1
	Positive predictive value, 95% CI							
Culture Karmali	100.0		96.4	88.2–99.2	95.4	84.5–98.9	96.3	88.0–99.2
Real-time PCR	77.0	66.5–87.6	94.2	84.2–98.1	93.6	83.0–97.9	94.9	86.2–98.5
Ridascreen^®^	69.7	59.4–80.1	86.9	77.0–93.3	86.5	75.0–92.8	84.6	74.4–91.8
Premier^®^Campy	76.8	66.9–86.8	100.0	9.1–100.0	95.8	69.4–99.8	92.4	83.1–97.3
ImmunoCard Stat!^®^Campy	58.2	47.4–69.1	78.0	67.9–85.9	74.4	59.9–84.5	70.7	59.4–79.9
*P*	9.0	6.9–11.5	11.4	9.2–14.2	10.9	8.3–14.1	10.5	8.4–13.3
*σ*					0.9	0.5–1.2	1.7	1.0–2.4

95% CI, two-sided 95% confidence interval.

LCM CD, latent class model with a residual dependence common to all tests; LCM CI, latent class model under conditional independence; LCM SD, latent class model with a residual dependence specific to the three immunoenzymatic tests.

NE, not estimated because of estimate on the boundary.

*p*, prevalence of campylobacter infection.

*σ*, random effect.

We note that leave-one-test-out analyses did not show relevant differences in the prevalence and diagnostic accuracy when removing each immunoenzymatic medical tests one by one (Table 4).

**Table 4.** Diagnostic accuracy of medical tests according to leave-one-test-out analyses for LCM SD model, CampyLCA study, France, 2016

	without Ridascreen^®^		without Premier^®^Campy		without ImmunoCard Stat!^®^Campy	
	Sensitivity (95% CI)					
Culture Karmali	82.2	(69.8–90.2)	81.1	(70.0–89.7)	80.8	(70.5–89.0)
Real-time PCR	87.3	(76.9–93.9)	88.8	(78.5–95.0)	86.8	(77.0–93.2)
Ridascreen^®^			100.0	–	98.7	(57.1–98.6)
Premier^®^Campy	98.0	(2.4–99.9)			97.2	(55.9–97.6)
ImmunoCard Stat!^®^Campy	85.8	(14.5–95.3)	86.1	(73.8–92.9)		
	Specificity (95% CI)					
Culture Karmali	99.7	(98.7–99.9)	99.5	(98.5–99.8)	99.6	(98.7–99.9)
Real-time PCR	99.3	(98.2–99.8)	99.5	(98.5–99.8)	99.4	(98.4–99.8)
Ridascreen^®^			98.1	(95.9–99.0)	98.2	(56.7–98.0)
Premier^®^Campy	99.1	(4.0–99.8)			99.2	(57.8–99.2)
ImmunoCard Stat!^®^Campy	95.8	(9.5–97.5)	95.9	(93.0–97.3)		
*P*	10.6	(8.4–13.2)	10.5	(8.3–13.0)	10.7	(8.6–13.3)
*σ*	4.7	(−2.1 to –11.6)	1.2	(0.4–2.0)	0.0	(−10.3 to −10.6)

LCM SD, latent class model with a residual dependence specific to the three immunoenzymatic tests.

95% CI, two-sided 95% confidence interval.

*p*, prevalence of campylobacter infection.

*σ*, random effect.

## Discussion

Using a reference standard whose diagnostic accuracy is known to be far from perfection necessarily leads to biased estimations of new detection tests if the imperfection is not properly taken into account. Based on a dataset of 623 patient samples, the diagnostic accuracy of five tests of campylobacter infection was estimated by using LCMs to palliate the imperfection of the bacteriological culture. The model with a specific dependence within the three immunoenzymatic tests showed the best performances in terms of fit and compliance with LCM assumptions. The prevalence of infection was estimated about 10%. LCM results confirmed the moderate sensitivity and almost perfect specificity of the culture. Ridascreen^®^ and Premier^®^Campy showed very high sensitivities (98.5% and 97.2%, respectively) and very high specificities (97.9% and 99.1%, respectively) confirming their potential usefulness for diagnosing campylobacter infection in clinical practice.

As expected with the moderate sensitivity of culture, the prevalence of infection was higher when estimated by LCM, even if the difference remained tenuous. The incorrect use of culture as a reference standard also led to the underestimation of sensitivity and specificity of all the index tests. Indeed, when using a reference standard with moderate sensitivity and perfect specificity, as a culture, the patients falsely considered as disease-free contribute to wrongly classify positive results of index tests as ‘false positives’ and negative results as ‘true negatives’. For index tests with high sensitivity, this leads to a systematic underestimation of their sensitivity and specificity (as found in our application) and, to a greater extent, of their positive predictive value. In our campylobacter case, the use of bacteriological culture as a reference standard underestimated the positive predictive value of index tests from 12% to 18%.

Classically, LCMs rely on the assumption of conditional independence between tests. This hypothesis is implausible in many clinical situations but rarely evaluated in the literature while its violation may induce biased estimations of diagnostic accuracy [14]. It is, therefore, crucial to assess different structures of residual correlation and rely on the technical and biological mechanisms of index tests for the choice of the structures [38]. We proposed, for instance, a specific dependence between the three immunoenzymatic tests because of their common characteristics to detect campylobacter antigens. Assessing LCM models and their assumptions is not straightforward. We used different goodness-of-fit statistics and local residual dependence checking (pairwise graphs and testing) methods that were proposed in the literature. We showed that conclusions could vary according to the method, confirming the need to perform different checks in order to obtain a body of evidence on the adequacy of the model and ensure the credibility of the results. Indeed, all the tests rejected the adequacy of conditional independence and common dependence LCMs but while the global tests did not reject the adequacy of the specific dependence LCM, some bivariate statistics still rejected the local independence assumption at the level of 5%. One may question the power of global tests compared with the bivariate statistics. Yet, with our sample size, global tests using empirical distributions showed high capabilities to detect violation of conditional independence assumption with statistical power ranging from 87% to 95% according to a supplementary simulation study (Table 5).

**Table 5.** Statistical power of goodness-of-fit statistics (in %) using empirical distribution to detect violation of the conditional independence hypothesis when applying LCM CI model, CampyLCA study, France, 2016

True model	Pearson statistics	Likelihood ratio statistics	Power divergence statistics
LCM CD	86.6	92.4	91.8
LCM SD	93.4	95.0	95.0

LCM CI, latent class model under conditional independence; LCM CD, latent class model with a residual dependence common to all tests; LCM SD, latent class model with a residual dependence specific to the three immunoenzymatic tests.

Statistical power is defined as the percentage of times the test concludes that observations and predictions significantly differ (at a 5% significance level) when they actually do.

In our case example, three of the five diagnostic tests were immunoenzymatic tests (two ELISAs and one immunochromatographic test). We took into account the induced correlation with the specific random effect and this specification of LCM provided the best solution. However, the bigger weight of immunoenzymatic tests could have still influenced the diagnostic accuracy results estimated with LCM SD. To assess to which extent the results were influenced, we re-estimated the model by excluding one immunoenzymatic test at a time in a ‘leave-one-test out’ procedure. We found out that the point estimates were not much different although wider confidence intervals were obtained. In our case study, patients were recruited in two hospitals which could have induced a residual dependence within the hospital. We evaluated a potential impact of the study hospital on our results by adding a study hospital variable in the final LCM. The introduction of the study hospital did not significantly modify the estimation of prevalence of campylobacter (*β* = 0.29, *p* = 0.30) or diagnosis performances of the five tests (*β* = 0.34, *p* = 0.11).

Sparse data are almost inherent in diagnostic test evaluation due to the improvement of index tests and the limited sample sizes. Profiles with perfectly concordant responses (‘all positive’ and ‘all negative’) bring together almost all of the information (e.g. 90.4% in our application) while most of the discordant responses comprise no or a few observations only. As a result, asymptotic distributions of goodness-of-fit tests do not apply and empirical distributions under the null hypothesis have to be derived [23, 24, 36]. With the level of sparseness of our data, we confirmed the impaired type-I error rates of the statistics using asymptotic distributions with either too conservative (type I error rates down to 0.002) or anticonservative (type I error rates up to 0.116) tests (Table 6) while by definition, the type-I error remained correct when using the empirical distribution. In our application, this translated into discordant results between asymptotic and empirical distributions, especially for the model with specific dependence. We also observed that *P*-values resulting from different statistics were more consistent when using empirical distributions. The use of quantitative test results may solve the sparseness problem and may allow more precise specification by including covariates or multiple random effects for example. However, this would also require some reflection about how to summarise quantitative tests results and provide useful criteria for the clinical decision.

**Table 6.** Type-I error rates of goodness-of-fit statistics (in %) using asymptotic distribution for each model, CampyLCA study, France, 2016

Models	Pearson statistics	Likelihood ratio statistics	Power divergence statistics
LCM CI	11.6	0.2	2.4
LCM CD	6.2	1.4	1.8
LCM SD	8.0	0.2	3.6

LCM CI, latent class model under conditional independence; LCM CD, latent class model with a residual dependence common to all tests; LCM SD, latent class model with a residual dependence specific to the three immunoenzymatic tests.

Type I error rate is defined as the percentage of times the test concludes that observations and predictions significantly differ (at a 5% significance level) while they actually do not. The nominal value of type I error rate is 5%.

Beyond their use for diagnostic accuracy assessment, LCMs remain complex models that require specific attention. The likelihood of LCM is often multimodal so that algorithms may converge to local maxima. To ensure convergence to the global maximum of Likelihood, required for correct inference, multiple sets of initial values can be used (we considered 100 sets in our application). In addition, the inclusion of random effects to account for the possible residual dependency induces a numerical integration in the likelihood which highly complicates the estimation process and may also pose convergence problems, in particular in SAS Proc NLMIXED in our experience. For instance, in the LCM with a common dependence estimated with this procedure, a simulation study highlighted biased estimations with unacceptable parameter coverage rates while the procedure under R provided correct inference with no bias and acceptable coverage rates (results not shown).

Because the target condition does not rely on a clinical definition, LCMs can be considered as a black box and make clinicians feel uncomfortable about what the results represent [5, 20]. Moreover, the statistical classification may not fully coincide with pre-existing knowledge of the target condition or it may even refer to a related, but different condition [6, 38]. This approach becomes meaningful when all index tests included in the model rely on an established clinical and biological background and the condition definition is not ambiguous. That explains why LCMs are very popular in the infectious field where the condition is clearly defined (i.e., presence or absence of the bacteria) and where tests directly identify the presence of the microorganism or of its antigens or DNA [38]. Note that other approaches dealing with the imperfection of the reference standard (discrepant analysis, composite reference standard) have been highly criticised in the literature for not satisfying basic requirements of the diagnostic accuracy assessment [5, 39, 40].

A critical limit of LCM approach lies in the number of available index tests needed to implement the models: three tests for the basic model and more for extended ones. Some authors recommended the use of at least 10 tests to ensure the distinction between different correlation structures [19]. In our context, with five tests, we did find differences between the LCMs structures hereby reported. Note that other LCMs structures, which performed worse than the specific dependence model, are not reported.

From a practical point of view, our feeling is that a major current drawback of LCM techniques for diagnostic accuracy assessment lies in the gap between recommendations that advise a search for specific correlation structure, posterior evaluation, goodness-of-fit statistics and graphs and programs available in standard software [14, 15, 19, 22–24, 36–38]. Model specifications are relatively limited, the correct convergence of models is not systematically ensured and a posteriori evaluation usually requires programming skills, which reduces the applicability of LCMs in the clinical epidemiology community.

In conclusion, the imperfection of the reference standard precludes the valid estimation of diagnostic accuracy parameters of new tests using standard methods and no good solution has been proposed so far. LCMs constitute a promising way to overcome it, on the condition that they are correctly specified and assessed [14]. However, this technique still requires substantial developments in usual software in particular to become a veritable solution for statisticians or epidemiologists involved in clinical epidemiology research.
